# Effect of phase-specific dietary black soldier fly larvae supplementation on growth performance and blood biochemical indices of local breed F2 meat roosters

**DOI:** 10.1016/j.psj.2026.106615

**Published:** 2026-02-10

**Authors:** Bin Wang, Baobei Ge, Yunzhi Peng, Feng Chen, Huize Tan, Xiaona Wei

**Affiliations:** a School of life science, Zhengzhou University, Zhengzhou 450001, China; b Wen's Group Academy, Wen's Foodstuffs Group Co., Ltd., Yunfu 527400, China; c College of Animal Science, South China Agricultural University, Guangzhou 510642, China

**Keywords:** Black soldier fly larvae, Phase-specific supplementation, Growth performance, Blood biochemistry indices, Native chicken

## Abstract

Black soldier fly larvae (BSFL, *Hermetia illucens*) have emerged as a promising alternative protein sources for livestock and poultry feed due to their high protein digestibility and environmental friendliness. However, most studies focus on full-cycle BSFL supplementation, with limited evidence for phase-specific effects in native chickens. This study investigated the impacts of 2% and 4% BSFL supplementation during the chick (1–25 days), grower (26–50 days), or finisher (51–70 days) stage on growth performance and blood biochemical indices of Local Breed F2 Meat Roosters. A total of 3150 1-day-old roosters were randomly assigned to 7 groups (6 replicates/group, 75 birds/replicate). Growth performance was monitored at each stage, and blood biochemical indices were measured at the end of trial. Results showed that supplementation with 2% BSFL during the chick stage increased average daily gain (ADG) and decreased the feed conversion ratio (FCR), while 4% BSFL supplementation during the finisher stage reduced FCR. Both treatments were beneficial to improving growth performance. In contrast, 4% BSFL supplementation during the chick stage increased mortality rate significantly compared to control group. Phase-specific BSFL supplementation significantly altered serum total protein (TP), lipoprotein levels, liver function, and metabolic enzyme activities. Specifically, 2% or 4% BSFL supplementation enhanced TP synthesis and regulated lipid metabolism indices. In conclusion, dietary supplementation with 2% BSFL during the chick stage is an optimal strategy to improve growth performance and metabolic health of native chickens, providing practical guidance for sustainable poultry production.

## Introduction

The animal husbandry industry, a core for global protein supply, has long faced protein feed shortages. As a major livestock producer, China has expanded its industry scale in recent years, with growing feed output and value (China Feed Industry Association, 2025). However, key protein sources like soybean meal (SBM) and fish meal rely heavily on imports, with volatile prices due to global supply-demand and trade policies, destabilizing the industrial chain. Compounded by limited arable land (restricting soybean production), declining global fisheries (shrinking fish meal), and frequent livestock diseases, feed protein imbalance has worsened, urgently requiring low-cost, high-quality, sustainable alternatives (Van Huis *et al*., 2023).

Among alternatives, insects excel as green feed due to high protein, low emissions, and efficient resource recycling. The black soldier fly larvae (BSFL, *Hermetia illucens, Diptera: Stratiomyidae*) is a research hotspot, with unique traits and potential. A widely distributed resource insect (Surendra *et al.*, 2020), BSFL digest diverse organic wastes (manure, food waste, by-products) into high-quality protein and fat, with short cycles (≈30–45 days), small land use, and high conversion rates (20%–30% dry matter) (Diener *et al.*, 2009), fitting circular economies. Nutritionally, BSFL meal varies by substrate: protein 31.7%–47.6%, fat 11.8%–34.3% (Wang *et al*., 2017), post-drying/defatting, protein reaches 55.9%–63.9% on par with fish meal (Surendra *et al*., 2016), with balanced amino acids and functional components (calcium, lauric acid [40%–50% of fatty acids], antimicrobial peptides), qualifying it to replace conventional feeds. The European Union officially approved the use of insect-processed animal protein in poultry and pig feeds in 2021 further supports large-scale BSF use.

Current research on BSFL in livestock shows progress. In broilers, BSFL meal provides good energy and digestible amino acids, and BSFL fat replacing soybean oil can improve growth performance, carcass traits, and meat quality of chickens (Schiavone *et al*., 2017). BSFL-oil feeds outperform corn and coconut oil in feed conversion (Kim *et al.*, 2020). As a protein substitute, BSFL meal replacing partial SBM in broiler and quail diets matches control digestibility and efficiency (Barbi *et al.*, 2020, Bejaei *et al.*, 2018). Full or partial SBM replacement in layer diets causes no harm (Heuel *et al*., 2021), but reduces broiler intake, weight gain, and carcass quality (Murawska *et al.*, 2021). High-level (50%–100%) full-fat BSFL meal in broiler diets impairs growth and carcass quality (Murawska *et al.*, 2021). Additionally, a meta-analysis shows that replacing more than 10% of the diet with insects can reduce poultry weight gain (Moula *et al.*, 2019). Notably, BSFL’s medium-chain fatty acids (Kim *et al*., 2022) and antimicrobial peptides (Józefiak *et al.*, 2017, Park *et al.*, 2015) boost antioxidant and immune function by enhancing serum enzymes (Dabbou *et al*., 2018), bacterial clearance and improve the survival of broiler chicks (Lee *et al*., 2018), altering gut microbiota and short-chain fatty acids (SCFAs) to promote growth (Cutrignelli *et al*., 2018, Jin *et al.*, 2021).

However, most BSFL studies focus on full-cycle feeding, with little on stage-specific effects in broilers. Native breeds (e.g., Local Breed F2 Meat Roosters) have distinct growth traits, such as slower growth and higher environmental adaptability, compared to commercial broilers, implying different nutritional requirements across stages. Thus, this study aimed to address these gaps by investigating the effects of phase-specific (chick, grower, finisher) and dose-dependent (2%, 4%) BSFL supplementation on growth performance and blood biochemical indices of Local Breed F2 Meat Roosters. The findings provide a stage-adapted feed optimization strategy for native chicken production.

## Materials and methods

### Animals and Housing

A total of 3150 1-day-old Local Breed F2 Meat Roosters were purchased from Wens Foodstuff Group Co., Ltd. and reared at the Liangdong Broiler Experimental Farm of the Research Institute of Wens Foodstuff Group Co., Ltd. Chickens were randomly divided into 7 groups with 6 replicate groups per treatment group and 75 birds per replicate and housed in independent poultry houses. The experimental period lasted 70 days, with consistent rearing management across groups. Feed supply, drinking water provision, environmental cleaning, disinfection, and disease prevention measures were strictly implemented in accordance with the standard operating procedures of Wens Foodstuff Group Co., Ltd. During the brooding period, heat lamps were used to regulate the house temperature, which was maintained at 35°C in the first week and then gradually decreased by 2∼3°C per week until reaching the ambient temperature. Continuous lighting was provided during the brooding period, and the light duration was adjusted after the second week; good cross-ventilation in the poultry houses was maintained throughout the experiment.

The BSFL used in this study was provided by UNIQUE Co., Ltd (Guangzhou, China), with the following nutritional composition: 2.77% moisture, 33.1% crude protein, 30.0% crude fat, 5.13% calcium, 1.16% phosphorus, 19.8% crude ash, 1.93% lysine, 0.74% methionine and cystine, and 0.96% threonine.

### Experimental design

The grouping design was as follows: Group 1 served as the control group and was fed a basal diet predominantly composed of corn and soybean meal throughout the 70-day experimental period. Groups 2 and 3 were assigned to the chick stage (1–25 days of age) treatment, with the basal diet supplemented with 2% and 4% dried BSFL, respectively, during this stage; the basal diet alone was provided for the remaining growth periods. Groups 4 and 5 were the grower stage (26–50 days of age) treatment groups, where the basal diet was supplemented with 2% and 4% dried BSFL, respectively, during the grower stage, and the basal diet without BSFL was fed for all other stages. Groups 6 and 7 were allocated to the finisher stage (51–70 days of age) treatment, with 2% and 4% dried BSFL added to the basal diet during this stage, respectively, and the basal diet alone provided for the preceding growth periods. The basal diet used in the experiment was formulated with reference to the broiler feeding standards of Wens Foodstuff Group Co., Ltd. The composition, nutritional levels and Fatty acid (FA) compositions of the diet for each group at different stages is shown in Table 1, Table 2.

**Table 1:** Ingredients composition (g/kg), Nutritional Levels (%) of Diets for Each Group at Different Stages.

Item	Chick-stage (1∼25 days of age)			Grower-stage (26∼50 days of age)			Finisher-stage (51∼70 days of age)		
	Control (Group 1)	2% BSFL (Group 2)	4% BSFL (Group 3)	Control (Group 1)	2% BSFL (Group 4)	4% BSFL (Group 5)	Control (Group 1)	2% BSFL (Group 6)	4% BSFL (Group 7)
Corn (Dried, C-D Grade)	592.65	599.74	606.84	649.37	656.36	663.55	664.85	671.95	679.03
Soybean Meal (46% CP)	180.83	164.16	147.49	90.11	73.44	56.78	68.20	51.53	34.87
Canola Meal (36% CP)	80.00	80.00	80.00	80.00	80.00	80.00	80.00	80.00	80.00
Cottonseed Meal (46% CP)	30.00	30.00	30.00	50.00	50.00	50.00	50.00	50.00	50.00
Corn Gluten Meal (60% CP)	30.00	30.00	30.00	30.00	30.00	30.00	30.00	30.00	30.00
Sunflower Seed Meal	30.00	30.00	30.00	30.00	30.00	30.00	30.00	30.00	30.00
Grade 4 Soybean Oil	18.20	10.40	2.70	29.60	21.90	14.20	39.60	31.90	24.10
Calcium Carbonate	14.90	12.60	10.20	15.50	13.20	10.80	15.30	13.00	10.70
Lysine Sulfate (70% Active Ingredient)	4.74	4.94	5.14	7.05	7.25	7.45	6.41	6.61	6.81
Dicalcium Phosphate	8.70	8.00	7.20	6.50	5.80	5.00	4.90	4.10	3.40
0.4% Compound Enzyme (Huangxiao 111)	4.00	4.00	4.00	4.00	4.00	4.00	4.00	4.00	4.00
Sodium Chloride	3.20	3.20	3.30	3.30	3.30	3.30	3.30	3.30	3.30
Methionine (98% Purity)	1.09	1.16	1.22	2.25	2.32	2.38	1.40	1.46	1.53
Threonine (98% Purity)	0.49	0.60	0.71	1.42	1.53	1.64	1.24	1.35	1.46
Choline Chloride (60% Content)	0.80	0.80	0.80	0.50	0.50	0.50	0.40	0.40	0.40
Mold Inhibitor	0.4	0.4	0.4	0.4	0.4	0.4	0.40	0.40	0.40
BSFL	/	20.00	40.00	/	20.00	40.00	/	20	40
Nutritional Level (%)									
Moisture	12.63	12.51	12.46	12.09	11.99	11.84	11.65	11.59	11.52
Crude protein	20.3	20.44	20.39	17.26	17.73	17.64	16.89	16.44	16.57
Crude ash	4.75	4.97	4.83	4.25	4.35	4.26	4.02	3.88	3.82
Crude fat	3.51	3.31	3.3	4.76	4.59	4.35	5.41	5.3	5.29
Calcium	0.93	0.95	0.93	0.87	0.87	0.89	0.82	0.78	0.76
Total phosphorus	0.56	0.57	0.57	0.52	0.53	0.51	0.5	0.49	0.48

Note. The premix includes Vitamin A, Vitamin D3, Vitamin E, Vitamin K, Vitamin B1, Vitamin B2, Vitamin B6, Vitamin B12, calcium pantothenate, nicotinamide, folic acid, biotin, choline, copper, zinc, manganese, iron, iodine, selenium, and phytase. For the specific addition amounts, please refer to Chicken Feeding Standards (NY/T 33-2004).

**Table 2:** Fatty acid composition (% of total identified fatty acids) of Diets for Each Group at Different Stages.

FA (%)	Chick-stage (1∼25 days of age)			Grower-stage (26∼50 days of age)			Finisher-stage (51∼70 days of age)		
	Control (Group 1)	2% BSFL (Group 2)	4% BSFL (Group 3)	Control (Group 1)	2% BSFL (Group 4)	4% BSFL (Group 5)	Control (Group 1)	2% BSFL (Group 6)	4% BSFL (Group 7)
Capric acid (C10:0)	0.03	0.17	0.22	0.00	0.16	0.13	0.03	0.09	0.17
Lauric acid (C12:0)	0.30	3.38	5.52	0.08	4.56	2.29	0.38	2.30	4.99
Myristic acid (C14:0)	0.26	0.82	1.34	0.17	1.11	0.58	0.28	0.61	1.13
Pentadecanoic acid (C15:0)	0.03	0.04	0.06	0.03	0.05	0.04	0.02	0.03	0.04
Palmitic acid (C16:0)	14.86	15.52	16.85	14.09	15.60	14.28	13.43	13.89	14.82
Palmitoleic acid (C16:1)	0.27	0.52	0.76	0.21	0.58	0.44	0.21	0.36	0.53
Heptadecanoic acid (C17:0)	0.13	0.15	0.16	0.13	0.15	0.14	0.12	0.13	0.13
Stearic acid (C18:0)	3.47	3.30	3.12	3.61	3.35	3.26	3.73	3.41	3.27
Oleic acid (C18:1n9c)	22.00	22.05	22.43	23.27	22.64	23.39	23.96	23.61	23.49
Linoleic acid (C18:2n6c)	52.98	49.26	45.72	52.63	47.16	50.12	51.84	50.17	46.63
Arachidic acid (C20:0)	0.39	0.35	0.32	0.42	0.35	0.38	0.43	0.40	0.38
α-Linolenic acid (C18:3n3)	4.31	3.53	2.52	4.39	3.40	3.92	4.43	3.93	3.38
cis-11-Eicosenoic acid (C20:1)	0.23	0.22	0.19	0.25	0.22	0.27	0.27	0.26	0.24
Heneicosanoic acid (C21:0)	0.00	0.00	0.00	0.00	0.00	0.00	0.03	0.02	0.02
Eicosadienoic acid (C20:2)	0.02	0.00	0.00	0.00	0.00	0.00	0.04	0.04	0.04
Behenic acid (C22:0)	0.32	0.26	0.21	0.38	0.27	0.33	0.39	0.34	0.31
cis-Arachidonic acid (C20:4n6)	0.02	0.06	0.12	0.00	0.00	0.04	0.00	0.00	0.05
cis-Erucic acid (C22:1n9)	0.02	0.02	0.02	0.02	0.02	0.02	0.05	0.05	0.05
Tricosanoic acid (C23:0)	0.06	0.05	0.05	0.06	0.05	0.06	0.06	0.06	0.05
Eicosapentaenoic acid (C20:5n3)	0.02	0.07	0.15	0.02	0.10	0.07	0.01	0.04	0.06
Lignoceric acid (C24:0)	0.26	0.23	0.23	0.26	0.23	0.24	0.25	0.25	0.22

### Data collection

At the start of each experimental stage, the initial body weight of broilers in each group was measured; at the end of each stage, the final body weight was recorded (feed was withdrawn on the night before weighing, while ad libitum access to drinking water was maintained during feed withdrawal). Daily records were kept for feed intake, number of dead and culled birds, weight of dead and culled birds, as well as the occurrence of other diseases and corresponding treatment measures in each group. Growth performance indices such as average daily gain (ADG) and feed conversion ratio (FCR) were calculated based on each replicate group.

At the end of experiment, 12 healthy broilers from each group were randomly selected for blood sampling via brachial vein puncture. Blood was collected in tubes without an anticoagulant. Serum was collected after centrifugation at 3,000 × g for 10 min and subjected to analyze biochemical indices.

### Statistical analysis

All experimental data were expressed as mean ± standard deviation (SD) and analyzed using GraphPad Prism software (version 9.0.0). One-way analysis of variance (ANOVA) was used to compare differences among groups. *P*-value < 0.05 was considered statistically significant.

## Results

### Effects of BSFL Supplementation on growth performance of chickens

Table 3 shows the effects of different dietary doses of BSFL on the growth performance of native chickens at different growth stages. Dietary supplementation of 2% BSFL at the chick stage (Group 2) significantly increased the ADFI and ADG of chicks and decreased the FCR. Although the ADFI of chickens in this treatment group decreased and increased at the grower and finisher stages, respectively, there was no significant change in the overall ADFI and ADG throughout the entire feeding period, but the FCR showed a decreasing trend. Supplementation of 2% or 4% BSFL at the grower and finisher stages had no significant effect on the growth of local chickens. However, supplementation of 4% BSFL at the chick stage significantly increased the culling rate of the flock. In summary, monitoring of the growth performance of local breed chickens at each growth stage and the entire cycle indicated that dietary supplementation of 2% BSFL at the chick stage is beneficial to improving their growth performance.

**Table 3:** Effects of different dietary doses of BSFL on growth performance of native chickens at different growth stages.

	Chick stage (1-22d)				Grower stage (22-42d)				Finisher stage (43-63d)			
BSFL Supplementation	Group 1	Group 2	Group 3	P value	Group 1	Group 4	Group 5	P value	Group 1	Group 6	Group 7	P value
	0	2%	4%		0	2%	4%		0	2%	4%	
Chick stage (1-22d)												
ADFI (g/day/bird)	24.3±0.2b	24.9±0.3a	24.9±0.2a	0.047								
ADG (g/day/bird)	14.3±0.2b	14.9±0.2a	14.6±0.1ab	0.022								
FCR (kg/kg)	1.70±0.01ab	1.67±0.01b	1.71±0.01a	0.029								
Survival rate (%)	99.5±0.5	98.7±0.4	97.6±0.6	0.089								
Grower stage (22-42d)												
ADFI (g/day/bird)	69.3±1.3ab	65.2±1.1b	71.4±1.5a	0.016	69.3±1.3	70.4±1.8	68.3±1.4	0.6088				
ADG (g/day/bird)	26.0±0.5	26.3±0.5	25.9±0.5	0.883	26.0±0.5	25.9±0.3	26.8±0.6	0.3414				
FCR (kg/kg)	2.67±0.10	2.48±0.05	2.77±0.05	0.06	2.67±0.10	2.71±0.06	2.55±0.07	0.332				
Survival rate (%)	99.7±0.3	99.2±0.3	98.2±1.1	0.464	99.7±0.3	99.3±0.3	99.3±0.3	0.5659				
Finisher stage (43-63d)												
ADFI (g/day/bird)	108±1ab	110±1a	105±1b	0.0177	108±1	106±1	106±1	0.1644	108±1	105±1	107±1	0.2354
ADG (g/day/bird)	37.1±0.6	37.5±0.4	35.9±0.6	0.0932	37.1±0.6	36.2±0.7	36.3±0.4	0.5306	37.1±0.6	36.1±0.6	37.0±0.4	0.564
FCR (kg/kg)	2.92±0.03	2.93±0.02	2.92±0.02	0.9908	2.92±0.03	2.92±0.03	2.92±0.03	0.9839	2.92±0.03	2.92±0.03	2.89±0.02	0.6205
Survival rate (%)	99.5±0.3	99.1±0.6	98.8±0.6	0.6812	99.5±0.3	99.5±0.3	99.5±0.5	0.9273	99.5±0.3	98.6±1.4	98.6±0.6	0.3974
Entire rearing period (1-63d)												
ADFI (g/day/bird)	69.2±0.1	68.2±0.9	68.1±1.4	0.289	69.2±0.1	68.5±0.7	68.0±0.6	0.0828	69.2±0.1	66.8±1.2	68.9±0.6	0.1056
ADG (g/day/bird)	26.0±0.1	26.4±0.3	25.8±0.1	0.1697	26.0±0.1ab	25.7±0.2b	26.0±0.2ab	0.4003	26.0±0.1	25.8±0.2	26.3±0.2	0.1039
FCR (kg/kg)	2.66±0.03ab	2.57±0.01b	2.70±0.02a	0.035	2.66±0.03a	2.66±0.02a	2.63±0.02ab	0.5621	2.66±0.03a	2.67±0.03a	2.62±0.02ab	0.2321
Survival rate (%)	98.7±0.7a	97.1±0.7ab	94.7±1.2b	0.027	98.7±0.7	98.0±0.5	98.0±0.5	0.6315	98.7±0.7	96.4±1.2	97.6±0.8	0.2974

Note. IBW- Initial body weight; FBW- Final body weight; ADFI- Average daily feed intake; ADG- Average daily gain; FCR- Feed-to-gain Ratio. Data are presented as mean ± standard deviation (SD). The P-values refer to the significance analysis for intergroup comparisons within the same growth stage. Different lowercase letters in the same row indicate significant differences among groups (P < 0.05).

### Effects of BSFL on serum biochemical indicators of broilers

At the end of the entire experiment, blood samples were collected from chickens in each group. Table 4 presents the effects of BSFL addition at different doses and stages on the serum biochemical indicators of local breed chickens in adulthood. At the finisher stage, a positive correlation was observed between dietary BSFL addition level and blood glucose concentration (Groups 6–7), indicating that BSFL supplementation can instantly improve the energy metabolism level of chickens. Results of protein metabolism indicators showed that dietary supplementation of 2% BSFL at the chick stage (Group 2) significantly increased serum total protein (TP) and globulin (GLB) concentrations at the end of the experiment; supplementation of 4% BSFL at the grower stage (Group 5) significantly increased serum TP, albumin (ALB), and GLB concentrations, while no significant change in albumin/globulin ratio (A/G) was observed after BSFL supplementation at any stage. Lipid metabolism related results showed that dietary supplementation of 2% BSFL at the chick stage (Group 2) significantly increased serum low-density lipoprotein cholesterol (LDL-C) concentration and decreased high-density lipoprotein cholesterol (HDL-C) concentration in local chickens. Dietary supplementation of 2% BSFL at the chick stage (Group 2) significantly increased serum total bile acid (TBA) concentration, while supplementation of 4% BSFL (Group 3) increased serum GGT level. Supplementation of 4% BSFL at the grower stage (Group 5) increased serum GGT level and decreased total bilirubin (TBIL) and indirect bilirubin (IBIL) concentrations. Similarly, supplementation of 4% BSFL at the finisher stage (Group 7) increased serum GGT level and decreased serum TBIL concentration. Poultry are uricotelic animals, and uric acid (UA) and creatinine (Cr) concentrations are core indicators evaluating avian renal function. Dietary supplementation of 2% BSFL at the chick stage (Group 2) decreased serum Cr concentration, while supplementation of 2% BSFL at the finisher stage (Group 6) decreased serum UA concentration. Glycylproline dipeptidyl aminopeptidase (GPDA), lactate dehydrogenase (LDH), creatine kinase (CK), and amylase (AMY) are tissue-specific enzymological indicators. Supplementation of 2% BSFL at the grower and finisher stages significantly decreased GPDA and AMY levels at the end of the experiment, respectively.

**Table 4:** Determination results of blood biochemical indicators.

Indicators	Chick-stage (1∼25 days of age)				Grower-stage (26∼50 days of age)				Finisher-stage (51∼70 days of age)			
	Control (Group 1)	2% BSFL (Group 2)	4% BSFL (Group 3)	P value	Control (Group 1)	2% BSFL (Group 4)	4% BSFL (Group 5)	P value	Control (Group 1)	2% BSFL (Group 6)	4% BSFL (Group 7)	P value
Glu	12.4±0.3	12.9±0.3	13.1±0.4	0.285	12.4±0.3	12.6±0.4	13.0±0.4	0.556	12.4±0.3b	12.8±0.2ab	13.5±0.3a	0.032
TP	37.4±0.6b	40.9±0.7a	37.5±1b	0.006	37.4±0.6b	37.0±0.7b	45.0±1.4a	<0.0001	37.4±0.6	40.2±1.5	38.3±0.9	0.215
Alb	13.6±0.5	14.8±0.4	13.3±0.5	0.093	13.6±0.5b	13.5±0.3b	15.2±0.4a	0.011	13.6±0.5	14.2±0.4	14.5±0.5	0.358
Glo	23.5±0.7b	27.0±1a	24.9±0.6ab	0.016	23.5±0.7b	23.7±0.6b	29.8±1.3a	0.001	23.5±0.7	26±1.2	24.1±0.7	0.148
A/G	0.56±0.02	0.55±0.02	0.53±0.02	0.446	0.56±0.02	0.56±0.02	0.52±0.02	0.219	0.56±0.02	0.55±0.02	0.59±0.02	0.287
TG	0.54±0.03	0.62±0.03	0.63±0.06	0.2678	0.54±0.03	0.47±0.02	0.53±0.03	0.071	0.54±0.03	0.46±0.02	0.49±0.02	0.050
TC	3.76±0.2	4.02±0.15	3.69±0.17	0.384	3.76±0.2	3.78±0.14	3.99±0.17	0.601	3.76±0.2	3.82±0.11	4.16±0.15	0.171
HDL-C	2.37±0.14	2.32±0.09	2.36±0.1	0.946	2.37±0.14	2.56±0.08	2.46±0.11	0.471	2.37±0.14	2.48±0.09	2.74±0.13	0.105
LDL-C	1.08±0.07b	1.31±0.02a	1.10±0.08ab	0.041	1.08±0.07	1.17±0.06	1.33±0.08	0.072	1.08±0.07	1.22±0.09	1.18±0.06	0.416
HDL-C/LDL-C	2.22±0.13a	1.76±0.07b	2.22±0.15a	0.022	2.22±0.13	2.14±0.1	1.89±0.13	0.150	2.22±0.13	2.13±0.19	2.35±0.12	0.585
TC/HDL-C	1.58±0.03b	1.76±0.04a	1.56±0.04b	0.002	1.58±0.03	1.55±0.03	1.63±0.05	0.241	1.58±0.03	1.55±0.05	1.52±0.02	0.477
TBIL	2.47±0.22	1.94±0.21	1.88±0.13	0.072	2.47±0.22a	1.79±0.2ab	1.76±0.17b	0.027	2.47±0.22a	1.72±0.16b	2.37±0.21ab	0.024
DBIL	1.40±0.13	1.43±0.24	1.31±0.12	0.87	1.40±0.13	1.25±0.16	1.60±0.18	0.297	1.40±0.13	1.400±0.20	1.41±0.10	0.997
IBIL	0.9±0.22	0.51±0.17	0.57±0.13	0.402	0.9±0.22a	0.44±0.10ab	0.16±0.03b	0.022	0.9±0.22	0.32±0.11	0.84±0.25	0.168
TBA	5.38±0.60b	8.91±1.14a	7.65±0.80ab	0.030	5.38±0.60	7.81±1.44	4.72±0.75	0.097	5.38±0.60	4.51±0.46	5.84±0.73	0.311
AST	231±9	233±6	237±8	0.828	231±9	238±6	249±11	0.330	231±9	233±7	245±4	0.314
ALP	1916±377	2557±338	1767±185	0.203	1916±377	1656±88	2315±451	0.423	1916±377	2406±619	1850±296	0.676
GGT	21.4±1.2b	23.2±1b	29.7±2a	0.002	21.4±1.2b	21.6±1.2ab	26.4±1.5a	0.022	21.4±1.2b	23±0.8ab	27.9±1.9a	0.010
Cr	6.40±0.27a	6.56±0.34a	5.50±0.27b	0.036	6.40±0.27	5.80±0.18	6.50±0.37	0.055	6.40±0.27	5.70±0.26	6.00±0.30	0.224
Urea	0.23±0.04	0.23±0.01	0.21±0.02	0.772	0.23±0.04	0.21±0.02	0.24±0.02	0.760	0.23±0.04	0.23±0.02	0.23±0.02	0.995
UA	131±8	122±12	108±7	0.190	131±8	112±3	118±12	0.288	131±8a	103±5b	117±8ab	0.032
GGPDA	9.20±0.98	8.67±0.91	7.30±0.4	0.502	9.20±0.98a	5.93±0.21	6.67±0.24ab	0.022	9.20±0.98	7.10±0.55	6.60±0.27	0.161
LDH	1227±136	1493±205	1203±174	0.446	1227±136	1280±109	1237±103	0.941	1227±136	1457±131	1371±161	0.526
CK	1561±242	1343±118	1284±99	0.468	1561±242	1423±91	1524±136	0.828	1561±242	1238±114	1524±138	0.374
AMY	410±23	363±23	358±26	0.238	410±23	378±25	343±24	0.169	410±23a	293±20b	339±18ab	0.001

Note: Glu-Glucose, TP-Total Protein; Alb-Albumin; Glo-Globulin; TG-Triglycerides; TC-Total Cholesterol; HDL-C-High-Density Lipoprotein Cholesterol; LDL-C-Low-Density Lipoprotein Cholesterol; TBIL-Total Bilirubin; DBIL-Direct Bilirubin; IBIL-Indirect Bilirubin; TBA-Total Bile Acid; Cr-Creatinine; UA- Uric Acid; GGPDA- Glycylproline Dipeptidyl Aminopeptidase; AST- Aspartate Aminotransferase; ALP- Alkaline Phosphatase; GGT- Gamma-Glutamyl Transferase; LDH- Lactate Dehydrogenase; CK- Creatine Kinase; AMY- Amylase. Data are presented as mean ± SD. The P-values refer to the significance analysis for intergroup comparisons within the same growth stage. Different lowercase letters in the same row indicate significant differences among groups (P < 0.05).

## Discussion

BSFL have emerged as a research hotspot as an alternative protein source in livestock and poultry feed due to their high protein content, high digestibility, and environmental friendliness. However, studies on the stage-specific feeding effects of BSFL in native chicken breeds remain scarce. This study aimed to investigate the impacts of different growth stages and BSFL supplementation levels on the growth performance and blood biochemical indices of native chickens.

### Effects of BSFL on growth performance of native chickens

The chick stage is a critical period for the development of intestinal digestive function, immune organs, and metabolic systems in local breed chickens. At this stage, chickens have insufficient digestive enzyme secretion, immature intestinal barrier function, low nutrient utilization of the basal diet, and are prone to growth retardation and high mortality. Dietary fatty acid detection showed that BSFL supplementation significantly increased the content of medium-chain saturated fatty acids (lauric acid and capric acid) in the diet (Table 2). Medium chain fatty acids can be rapidly absorbed by the chick intestine without bile salt emulsification, which not only provides efficient energy substrates for the rapid growth of chicks but also exerts a natural antibacterial effect by damaging the cell membrane of intestinal pathogens (*Escherichia coli, Salmonella*), improving intestinal microecological balance, reducing nutrient absorption disorders caused by intestinal inflammation, thereby increasing feed utilization and decreasing FCR (Wu *et al.*, 2021). Notably, dietary supplementation of 4% BSFL at the chick stage significantly increased the culling rate of the flock. It is speculated that high-dose chitin in BSFL may stimulate the chick intestinal mucosa, damage the integrity of the intestinal barrier, and further affect nutrient absorption (Bovera *et al*., 2015, Marono *et al.*, 2017). This result is consistent with the findings of Hartinger *et al*. (2021), who reported that the ADG reached the peak when 15% defatted BSFL meal was used to replace soybean meal in the diet of commercial chicks, and the ADG significantly decreased when the replacement ratio exceeded 20%. Tykałowski *et al*. (2023) also found that when the chitin content of full-fat BSFL meal in the diet exceeded 5%, the crypt depth of the chick intestine increased, which is an important marker of intestinal damage, indirectly confirming the "intestinal damage effect" of 4% BSFL supplementation at the chick stage.

BSFL supplementation at the grower and finisher stages had no significant effect on growth performance. At the grower stage, the intestinal digestive function and liver metabolic function of chickens have matured, and the nutrient supply of the basal diet can meet their growth needs, so the nutritional supplement of BSFL did not show additional growth advantages. At the finisher stage, the growth of local breed chickens focuses on body fat deposition and meat quality formation, and their growth rate is much lower than that of fast-growing broilers. At this stage, the energy supply of feed is the core demand, and the protein and fatty acid supplements of BSFL did not change the core balance of energy metabolism, thus no significant growth-promoting effect was observed. This result is inconsistent with the study of Cullere *et al*. (2019), who found that replacing soybean oil with 10% BSFL fat in the diet of commercial broilers during the finishing period could increase ADG by 5.8% and decrease FCR by 4.3%. The BSFL supplementation level at the finisher stage of local chickens in this study was only 4%, much lower than that in the study of Cullere *et al*., which may be one of the reasons for the no significant change in growth performance.

Notably, a cross-stage persistence effect was observed in this experiment: chickens supplemented with 2% BSFL at the chick stage showed obvious advantages in growth performance in the middle and late feeding periods. It is speculated that supplementation of 2% BSFL at the chick stage can optimize intestinal morphology (Sjofjan *et al*., 2025), reflecting the long-term benefit of "early healthy intestinal foundation". And the intestinal damage caused by the 4% supplementation group is "irreversible", which will affect the nutrient absorption efficiency at the growing stage even if the high-dose supplementation is stopped.

Full-cycle data showed that the 2% supplementation group at the chick stage and the 4% supplementation group at the finishing stage had the highest weight gain. Notably, the FCR of the 2% supplementation group at the chick stage was significantly lower than that of the control group. The advantage of this "stage-adapted" supplementation scheme is that adding 2% BSFL at the chick stage can not only "lay the foundation" for intestinal development but also avoid the risk of high doses. This is consistent with the conclusion of Dorper *et al.* (2024), who reported that stage-specific supplementation of BSFL to slow-growing broilers could increase the full-cycle ADG by 7.8%.

### Metabolic regulatory mechanisms of BSFL on blood biochemical indices of native chickens

In this experiment, blood samples were only collected at the end of the feeding experiment to detect biochemical indicators. Therefore, the detection results of BSFL supplementation at the chick and grower stages reflect the long-term residual metabolic effect of the body after the end of supplementation, while the results of BSFL supplementation at the finisher stage are the immediate metabolic effect after the end of supplementation.

This study found a positive correlation between BSFL addition level and blood glucose concentration at the finisher stage, which reflects the immediate energy metabolism improvement effect of BSFL on chickens at the finisher stage. Glycogen, easily digestible carbohydrates, and fatty acids contained in BSFL can promote the absorption efficiency of carbohydrates in the chicken intestine, and simultaneously activate the hepatic gluconeogenesis pathway, providing sufficient energy substrates for body fat deposition and growth at the finisher stage (Park *et al.*, 2025). However, BSFL supplementation at the chick and grower stages had no significant effect on glucose concentration, because the detection results of these two stages are the long-term residual effect after supplementation, and the chicken body has achieved blood glucose balance through its own metabolic homeostasis regulation, without significant changes in indicators, which also reflects the metabolic adaptability of the chicken body to early nutritional intervention.

In this experiment, serum TP concentration was significantly increased in both the 2% supplementation group at the chick stage and the 4% supplementation group at the grower stage. This is consistent with the results of Alafif *et al.* (2025), who confirmed that the ileal digestibility of essential amino acids in BSFL can reach 85%∼90%, indicating that its protein can continuously promote body protein synthesis. The chick stage is a critical period for immune organ development, and the specific increase in globulin suggests that the high-quality protein in BSFL provides sufficient raw materials for the synthesis of immunoglobulins (IgG, IgM), realizing the immune improvement effect of early nutritional programming. Even after the end of supplementation, the body can still maintain a high reserve of immune proteins (Sjofjan *et al*., 2025). At the grower stage, the liver synthesis function of chickens has matured, and the simultaneous increase of albumin and globulin indicates that 4% BSFL supplementation at this stage can simultaneously improve the synthesis of nutritional proteins and immune proteins, which is a comprehensive regulation of protein metabolism (Dorper *et al*., 2024). The no significant change in A/G ratio indicates that BSFL supplementation at all stages did not break the balance between liver synthesis function and immune status, reflecting the safety of its protein nutrition regulation.

Changes in TG, cholesterol, and lipoprotein concentrations reflect the balance of lipid absorption, synthesis, and transport in the body. In this study, only 2% BSFL supplementation at the chick stage showed significant changes of increased LDL-C and decreased HDL-C. This result is not lipid metabolism disorder, but the physiological remodeling of lipid transport carriers induced by early nutritional intervention at the chick stage. Lipid transport in local breed chickens is mainly carried by HDL-C. After supplementation of 2% BSFL at the chick stage, the moderate increase of LDL-C can provide sufficient cholesterol for the development of peripheral tissues such as muscle and bone in chickens (Kim *et al.*, 2020), while the decrease of HDL-C is due to the rapid utilization of lipids by peripheral tissues rather than liver fat deposition. Combined with the results of significantly decreased FCR and improved growth performance in this treatment group, it can be confirmed that the changes of lipid indicators are physiological adjustments for the rapid growth of chicks, not pathological changes. Meanwhile, BSFL supplementation at all stages had no significant effect on TG and total cholesterol (TC), indicating that the regulation of BSFL on lipid metabolism of local breed chickens is mainly concentrated in the transport link, not in the absorption and synthesis links. This is related to the nutritional characteristics of BSFL that medium-short chain fatty acids are easily absorbed and have no risk of fat accumulation (Lau *et al*., 2024).

Bilirubin, TBA, and GGT are core indicators evaluating liver synthesis, excretion function, and biliary patency. This study found that appropriate dose of BSFL supplementation at each growth stage showed a benign regulatory effect on hepatobiliary function. Supplementation of 2% BSFL at the chick stage increased TBA, which can promote the emulsification and absorption of intestinal fat, improve the utilization rate of dietary lipids, and provide energy for chicken growth. Supplementation of 4% BSFL at different stages increased GGT level, and the moderate increase of GGT indicates the enhanced metabolic activity of hepatobiliary duct epithelial cells and optimized biliary excretion function, which is an important marker of improved liver metabolic function (Mahmoud *et al.*, 2025). The decrease of bilirubin (TBIL, IBIL) indicates that the liver's ability to decompose and metabolize hemoglobin, and bind and excrete bilirubin is improved, reducing the accumulation of bilirubin in the body and avoiding the damage of oxidative stress to the liver (Mahmoud *et al*., 2025). The differences in the regulation of hepatobiliary indicators by BSFL at different stages reflect the maturation of hepatobiliary function of chickens with growth and development. The regulatory effect at the growing and finishing stages is more comprehensive (increased GGT and decreased bilirubin), which also reflects that the regulation of BSFL on hepatobiliary function is development-dependent.

In terms of renal function indicators, supplementation of 2% BSFL at the chick stage decreased Cr, and supplementation of 2% BSFL at the finisher stage decreased UA. Cr mainly reflects the balance between muscle metabolism and renal filtration function. The decrease of Cr at the chick stage suggests that nutritional intervention with BSFL makes the muscle metabolism and renal filtration function of chicks more coordinated, reducing the accumulation of creatine metabolic waste. UA is the end product of protein metabolism in poultry. The decrease of UA at the finisher stage indicates that the high-quality protein in BSFL is easily digested and absorbed, reducing the production of endogenous nitrogen waste, reducing the nitrogen metabolic burden on the kidneys, and improving the renal filtration and excretion function (Dalmoro *et al.*, 2025). This result reflects the stage-specific protective effect of BSFL on the renal function of local breed chickens.

Tissue-specific enzymological indicators reflect the structural integrity and functional status of related organs in the body. This study found that supplementation of 2% BSFL at the grower stage decreased GPDA, and supplementation of 2% BSFL at the finisher stage decreased AMY. The decrease of these two indicators are benign physiological changes of the body, not organ function damage. GPDA is mainly distributed in the intestinal, liver, and kidney mucosal epithelial cells. The decrease of its blood content suggests that the antimicrobial peptides and medium-short chain fatty acids in BSFL play a protective role on mucosal epithelial cells, maintaining mucosal structural integrity and reducing enzyme release caused by mucosal damage. AMY is synthesized and secreted by the pancreas, and its decrease is a typical metabolic compensation effect (Seyedalmoosavi *et al*., 2022). BSFL supplementation at the finisher stage increased blood glucose level, and the body reduced pancreatic amylase secretion through negative feedback regulation, avoiding excessive decomposition of intestinal carbohydrates leading to high blood glucose, thereby maintaining blood glucose homeostasis. This change is not a decrease in digestive function, but a self-balance of body metabolic regulation.

### Limitations of this study

Although clear experimental results were obtained in this study, there are still some limitations. Firstly, Serum biochemical indicators were only detected by collecting blood samples at the end of the experiment, which cannot reflect the immediate metabolic changes and dynamic evolution of metabolic effects after BSFL supplementation at the chick and growing stages, making it difficult to determine the metabolic regulation peak of supplementation at each stage. Then, only two supplementation doses of BSFL (2% and 4%) were set in this study, which fail to determine optimal dose of BSFL at grower and fisher stage.

## Conclusions

Taking local breed F2 meat roosters as experimental subjects, this study explored the dose-specific regulatory effects of BSFL at three growth stages, and confirmed that 2% is the optimal supplementation dose at the chick stage. Meanwhile, combined analysis of growth performance and serum biochemical indicators revealed the synergistic regulation law of BSFL on the growth performance and body metabolism of local breed chickens, confirming that BSFL not only has a growth-promoting effect but also can realize the functional protection of multiple organs such as liver, kidney, hepatobiliary system, and intestine. This study provides a more comprehensive theoretical basis for the application of insect protein in local poultry breeding.

## CRediT authorship contribution statement

**Bin Wang:** Writing – review & editing, Project administration, Methodology, Investigation, Formal analysis. **Baobei Ge:** Writing – review & editing, Project administration, Data curation. **Yunzhi Peng:** Writing – review & editing, Investigation, Conceptualization. **Feng Chen:** Writing – review & editing, Supervision, Conceptualization. **Huize Tan:** Writing – review & editing, Supervision. **Xiaona Wei:** Writing – review & editing, Writing – original draft, Software.

## Disclosures

The authors declare that the research was conducted in the absence of any commercial or financial relationships that could be construed as a potential conflict of interest.
